# Preoperative state anxiety predicts postoperative health-related quality of life: A prospective observational study on patients undergoing lung cancer surgery

**DOI:** 10.3389/fpsyg.2023.1161333

**Published:** 2023-04-11

**Authors:** Shinnosuke Takamiya, Motoyasu Honma, Yuri Masaoka, Momoka Okada, Shinichi Ohashi, Yoko Tanaka, Kosuke Suzuki, Shugo Uematsu, Akihiko Kitami, Masahiko Izumizaki

**Affiliations:** ^1^Department of Physiology, Showa University School of Medicine, Tokyo, Japan; ^2^Respiratory Disease Center, Showa University Northern Yokohama Hospital, Yokohama, Japan

**Keywords:** QOL, STAI, state anxiety, trait anxiety, lung cancer surgery

## Abstract

**Objective:**

Improving quality of life (QOL) after surgery is very important. Recently, preoperative anxiety has been suggested to predict postoperative health-related (HR) QOL, however the accuracy of anxiety measurement remains problematic. We examined the relationship between preoperative anxiety level and postoperative HRQOL using qualitative and quantitative assessment of anxiety.

**Method:**

We used a detailed anxiety assessment to quantitatively investigate preoperative anxiety as a predictor of postoperative HRQOL in lung cancer patients. Fifty one patients who underwent surgery for lung cancer were included. They were assessed four times: on admission, on discharge, 1 month after surgery, and 3 months after surgery. Anxiety was measured separately as “state anxiety” and “trait anxiety” using the State–Trait Anxiety Inventory, and HRQOL was measured using the EuroQol 5 dimension 5-level.

**Results:**

The HRQOL decreased at discharge and gradually recovered over time, reaching the same level at 3 months after surgery as at admission. HRQOL score was lower at discharge than at pre-surgery and 3 months after the surgery (*p* < 0.0001 each), and the score at 1 month after the surgery was lower than at pre-surgery (*p* = 0.007). In addition, multiple regression analysis showed that HRQOL at discharge was associated with “state anxiety” rather than “trait anxiety” at admission (*p* = 0.004).

**Conclusion:**

This study identifies the types of anxiety that affect postoperative HRQOL. We suggest that postoperative HRQOL on discharge may be improved by interventions such as psychological or medication treatment for preoperative state anxiety if identified preoperative state anxiety can be managed appropriately.

## Introduction

As a surgeon, successful lung cancer surgery is not only about removing cancer; it is also essential in medical practice to respect the psychological aspects of the patient and to consider the prognosis of life (Batchelor et al., 2019). In particular, improving postoperative quality of life (QOL) is an important goal in clinical oncology, as is improving survival. For example, to improve health-related (HR) QOL, thoracic surgeons have attempted to establish less painful and less invasive surgical approaches such as video-assisted thoracoscopic surgery (VATS; Gonzalez et al., 2022). However, the results of studies on postoperative HRQOL are less consistent (Stamatis et al., 2019; Charles et al., 2021; Smolock et al., 2022), and controlling for postoperative HRQOL appears to be an extremely difficult task.

One reason for this is that postoperative HRQOL varies widely between individuals. It has been reported that pathological and technical factors, such as comorbidities, cancer stage, postoperative adverse events, and timing of chest tube removal, affect postoperative HRQOL (Ichimura et al., 2021a), and a variety of factors may be involved in postoperative HRQOL, increasing individual differences. Meanwhile, studies focusing on patients’ psychological aspects, which are factors other than pathological and technical factors in lung cancer surgery, have attracted attention. One study focused on preoperative anxiety and reported that it is a predictor of postoperative HRQOL (Ichimura et al., 2021b). A relationship between anxiety and HRQOL has traditionally been observed (Khoury et al., 2013; Hofmann et al., 2017; Carpenter et al., 2018), and attempts to predict postoperative HRQOL by preoperative anxiety have high feasibility and clinical relevance, as preoperative anxiety is likely to be reduced by psychological interventions such as therapy. However, in the previous study (Ichimura et al., 2021b), anxiety was assessed using a simple questionnaire with only one five-item question, which is insufficient for a qualitative and quantitative assessment of anxiety.

As anxiety can at least be divided into “state anxiety” and “trait anxiety” (Johnston, 1980; Suzuki et al., 2000), it is important to clarify which type of anxiety affects HRQOL. Furthermore, it is important to examine the relationship between anxiety and HRQOL quantitatively in order to make more accurate predictions. Because HRQOL immediately after surgery may be improved by interventions such as psychological or pharmacological treatment of preoperative state anxiety, if identified preoperative state anxiety can be managed appropriately. In the current study, we quantitatively examined the relationship between preoperative anxiety and postoperative HRQOL using an assessment from a specialized psychiatric unit that can assess anxiety from both state and trait aspects. Note that we did not interpret the patient’s type of anxiety as an anxiety disorder, but as normal response anxiety to surgery or illness. Therefore, no cut-off values for clinical diagnosis were established.

## Materials and methods

### Patients

**Figure 1:**
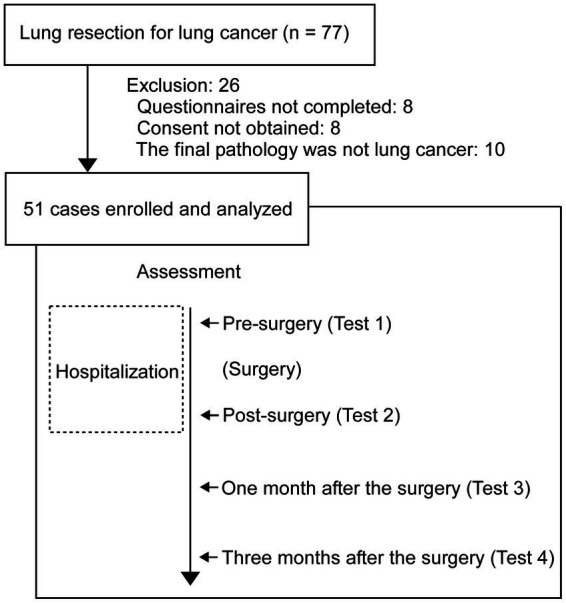
Patient flow diagram and time of assessment. The 51 patients were performed at 4 times for assessment (HRQOL, STAI, and pain survey). The background factor items were performed before pre-surgery.

This study was approved by the ethics committee of Showa University School of Medicine and conducted in accordance with the tenets of the Declaration of Helsinki (trial identifier number: 19H026). This study was registered in the University Hospital Medical Information Network (UMIN)-CTR (ID: UMIN000049049, 29/September/2022). We recruited 77 patients who underwent lung tumor surgery at Showa University Northern Yokohama Hospital from 19 October 2020 to 24 September 2021, and we collected data from 19 October 2020 to 14 December 2021. Of these 77, 51 patients were included in the analysis, excluding 8 cases with missing data (no questionnaires completed at admission or discharge), 8 cases in which the patient did not consent to the use of the data, and 10 cases in which the final pathology diagnosis was not lung cancer (Figure 1). The 51 patients provided written informed consent prior to the experiments. The authors had access to information that could identify individual participants during or after data collection.

### Study design

This was a prospective and observational study, and it was conducted in the single-center (Showa University Northern Yokohama Hospital, Yokohama, Japan). Tests were performed four times per patient. The first test was performed when the patient was admitted for surgery (Test 1), the second test was performed at the time of postoperative discharge (Test 2), the third test was performed 28 days after discharge (Test 3), and the fourth test was performed 84 days after discharge (Test 4; Figure 1). Follow-up was performed 112 days after discharge.

### Surgical treatment

The clinical path was used for the resection of the lung tumors. The chest tube was removed on postoperative day 3 (3-day pathway) and the patient was discharged on postoperative day 5 (5-day pathway). The mean length of stay was 7.06 days (standard deviation = 2.63). The chest tube drainage was a 20–24 Fr double lumen tube or a silastic tube (Satoh, 2016). Pain management included epidural analgesia and oral administration of a non-steroidal anti-inflammatory drug (Manion and Brennan, 2011). Epidural analgesia was tapered according to patient symptoms, and the epidural catheter was usually removed on the same day as the chest tube. If epidural analgesia was not indicated, intravenous analgesia was used.

To monitor perioperative clinical outcomes, adverse events (AEs) occurring during hospitalization were recorded according to the Japan Clinical Oncology Group (Clavien-Dindo classification) version 2.0 criteria for postoperative complications (grade I: Deviations from the normal postoperative course that do not require medical therapy or treatment by surgical, endoscopic or interventional radiology; grade II: Requires drug therapy other than antiemetics, antipyretics, analgesics, and diuretics; grade III: Requires surgical, endoscopic or IVR treatment; grade IV: Life-threatening complications and organ failure requiring quasi-intensive care unit/ICU management; grade V: Death of the patient; Katayama et al., 2016). Among the AEs recorded, grade 2 or higher were further classified. Pain and pleural effusion were not included in this study because they would have been grade 1 in all cases. Subcutaneous emphysema was documented by radiographs or examination findings.

### Assessments

Background factor items collected included age, sex, Charlson Comorbidity Index (Charlson et al., 1987), a lifetime smoking index calculated by multiplying the number of cigarettes per day by the number of years smoked, clinical stage according to the AJCC Cancer Staging Manual (8th edition), comorbidities (interstitial pneumonia, ischaemic heart disease, true diabetes mellitus, stroke, chronic obstructive pulmonary disease), and extent of lung resection. Additional data included length of postoperative stay, pathological stage according to the 8th edition of the tumor, node, metastasis staging system (Paner et al., 2018), and preoperative treatment (no patients were eligible in this case). The AEs version 2.0 criteria, which included whether adjuvant therapy was administered and postoperative adverse events that occurred during the hospital stay. Among the AEs recorded, events were further classified according to grade greater than 2.

As a clinical assessment, HRQOL was assessed using the EuroQol 5-Dimension-5-Level (EQ-5D-5L; Herdman et al., 2011; Norman et al., 2013). It consists of five dimensions: mobility, self-care, usual activities, pain/discomfort, and anxiety/depression. Each dimension is rated on a 5-point scale ranging from no problem (:1) to extreme problem (:5). Anxiety was measured using the State–Trait Anxiety Inventory (Johnston, 1980; Suzuki et al., 2000), which has been tested for reliability and validity (Oei et al., 1990; Moore et al., 1991). It is a self-administered questionnaire with 20 questions each on trait anxiety and state anxiety on a 4-point scale. Trait anxiety reflects a characteristic derived from one’s personality that tends to cause anxiety. State anxiety reflects a temporary anxious reaction to a particular time, scene, event, or object. In addition, pain at rest and during exercise was assessed using a visual analog scale.

### Statistical analysis

Repeated measures analysis of variance (RM-ANOVA) was used for trends in HRQOL scores (tests 1, 2, 3, and 4). The HRQOL score at discharge (test 2) was then used as the dependent variable. Clinical stage category (I, II, or higher), extent (partial resection or greater than or equal to area resection), surgical approach (VATS or open chest), lung resection mode [Wedge or other (Segmentectomy, Lobectomy, and Pneumonectomy)], sex, pain (at rest and on exertion) at admission and discharge, state and trait anxiety at admission and discharge, HRQOL at admission, maximum wound (cm), age, operation time, and blood loss were used as independent variables. The independent variables were analyzed individually using simple linear regression analysis. Then, from these independent variables, four variables that were significantly different and clinically significant were selected. The relationship between the independent variables and postoperative HRQOL (dependent variable) was examined by multiple linear regression analysis. To test the factor of type of surgery, two-way ANOVA was performed with the surgical approach and lung resection mode as factors for postoperative HRQOL. All tests were two-tailed. Statistical significance was set at adjusted *p*-values <0.05. SPSS version 26 for Windows (IBM, Inc., Chicago, IL) was used for analyses.

## Results

The background factor items are summarized in Table 1. State anxiety, trait anxiety, HRQOL scores, and pain (at rest and on exertion) are also summarized in Supplementary Table 1. Data without HRQOL assessment in postoperative tests 3 (*n* = 7) and 4 (*n* = 11) were treated as missing values. The mean age of the samples was 68.02 (S.D. = 10.091) and with male to female ratio of 31:18.

**Table 1:** Characteristics of patients.

Sex (male: female)		33:18
Age		68.02 ± 10.091
Stature (cm)		163.5 ± 8.48
Body weight (kg)		61.1 ± 10.4
Tumor diameter (mm)		17.4 ± 11.6
**CCI**		
	0	31 (60.8%)
	1	8 (15.7%)
	2	7 (13.7%)
	≥3	5 (9.8%)
	≥1	20 (39.2%)
**Comorbidities**		
	BA	3 (5.9%)
	Af	2 (3.9%)
	Hypertension	18 (35.3%)
	Interstitial pneumonia	1 (2%)
	Ischemic heart disease	3 (5.9%)
	Stroke	1 (2%)
	Diabetes mellitus	8 (15.7%)
	COPD	6 (11.8%)
Smoking index		578.5 ± 636.4
**Clinical stage category**		
	I	41 (80.4%)
	II	3 (5.9%)
	III	4 (7.8%)
	IV	1 (2.0%)
	≥II	8(15.7%)
Preoperative treatment		0
**Surgical approach**		
	Video-assisted thoracic surgery	17 (33.3%)
	Open chest	34 (66.7%)
**Lung resection mode**		
	Wedge	13 (25.5%)
	Segmentectomy	8 (15.7%)
	Lobectomy	28 (54.9%)
	Pneumonectomy	2 (3.9%)
	≥Segmentectomy	38 (74.5%)
**Postoperative adverse event, present**		
	All grades	32 (62.7%)
	≥Grade 2	7 (13.7%)
Operating time (min)		191.9 ± 79.9
Volume of blood loss (mL)		74.7 ± 182.9
Wound length (cm)		7.85 ± 3.81
Postoperative hospital stay (days)		7.06 ± 2.634
Postoperative treatment		13 (25.5%)

Values are presented as indicated or mean ± standard deviation or number of patients (%). BA, bronchial asthma; Af, atrial fibrillation; CCI, Charlson comorbidity index; COPD, chronic obstructive pulmonary disease.

**Figure 2:**
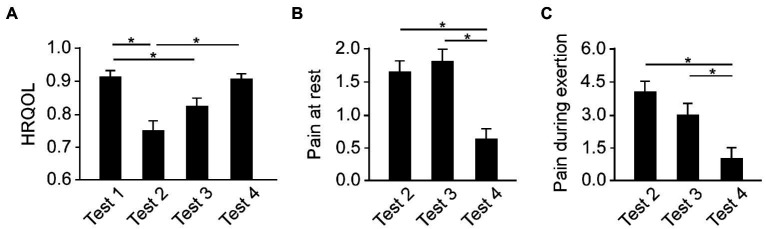
The transition of HRQOL and pain. **(A)** The HRQOL score was lowest at discharge (Test 2) and gradually recovered to the value at admission (Tests 3 and 4). **(B)** Pain at rest was lowest at 3 months after surgery (Test 4). **(C)** Pain during exertion was lowest at 3 months after surgery (Test 4). Error bars show the standard error of the mean. The asterisks mean a significant difference (*p* < 0.05). HRQOL, health-related quality of life.

First, the trend in HRQOL, pain at rest, and pain during exertion were analyzed using RM-ANOVA. The statistics revealed that the main effect of time course was significant (*F*_3,111_ = 18.387, *p* < 0.0001, *η*^2^ = 0.332). Post-hoc tests showed that HRQOL score was lower in test 2 than in test 1 and test 4 (*p* < 0.0001 each), and the score in test 3 was lower than in test 1 (*p* = 0.007; Figure 2A). In the trend in pain at rest, the RM-ANOVA revealed that the main effect of time course was significant (*F*_2,74_ = 5.799, *p* < 0.005, *η*^2^ = 0.135). *Post-hoc* tests showed that the pain at rest was lower in test 4 than in test 2 and test 3 (*p* < 0.05 each; Figure 2B). Similarly, in the trend on pain during exertion, the RM-ANOVA revealed that the main effect of time course was significant (*F*_2,74_ = 5.799, *p* < 0.005, *η*^2^ = 0.135). *Post-hoc* tests showed that pain during exertion was lower in test 4 than in test 2 and test 3 (*p* < 0.05 each; Figure 2C).

Second, a single regression analysis was performed separately with the HRQOL score in Test 2 as the dependent variable and 40 other variables (background factor items and clinical assessments) as independent variables (*n* = 51; Supplementary Table 2). Significant associations were found for comorbid stroke (*B* = −0.327, *p* = 0.019), adjuvant chemotherapy (*B* = −0.294, *p* = 0.037), state anxiety in test 1 (*B* = −0.440, *p* = 0. 001), HRQOL in test 1 (*B* = 0.287, *p* = 0.041), HRQOL in test 3 (*B* = 0.442, *p* = 0.003), pain at rest in test 2 (*B* = −0.342, *p* = 0.014) and pain on exertion in test 2 (*B* = −0.454, *p* = 0.001).

**Figure 3:**
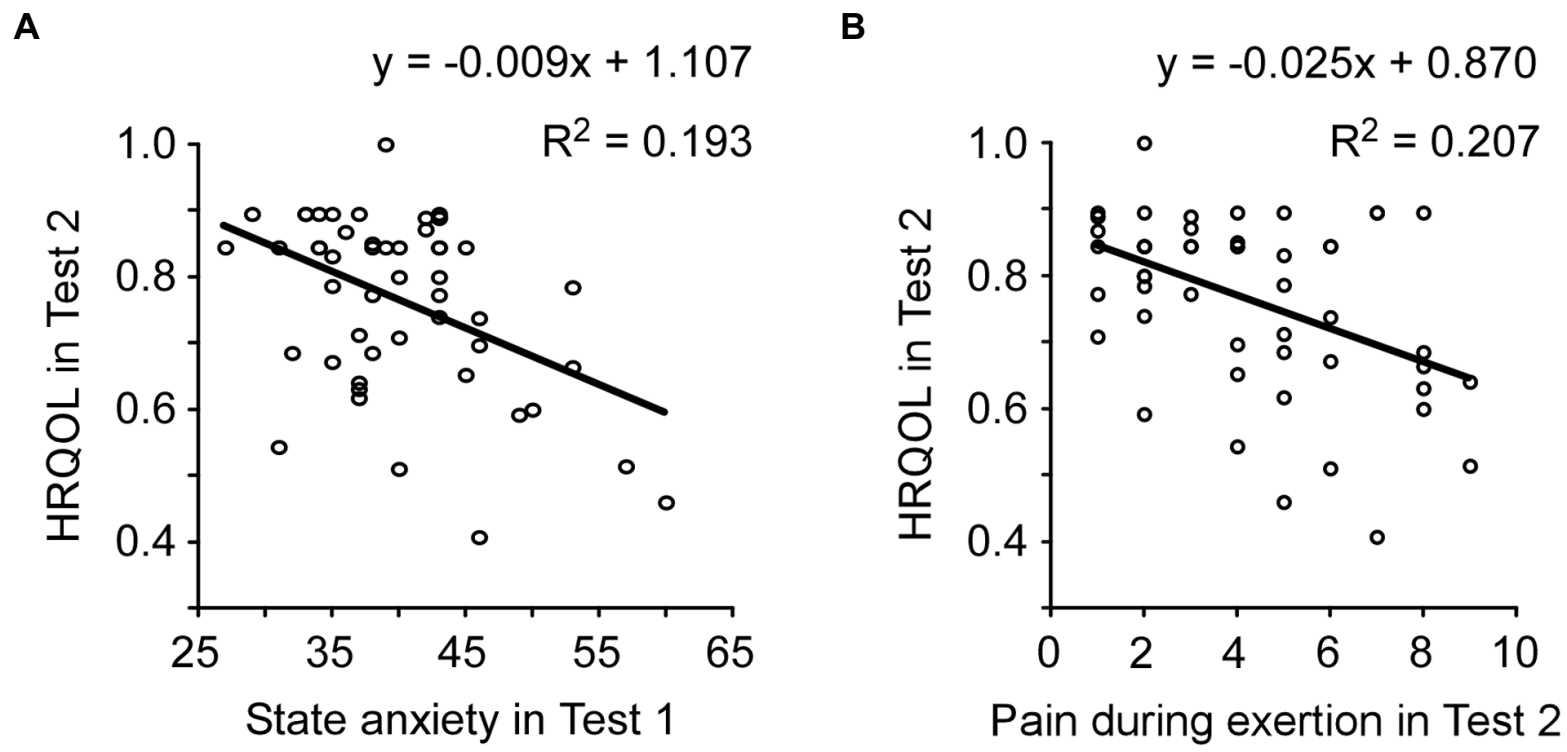
Factors associated with postoperative HRQOL. **(A)** State anxiety in preoperative (Test 1) was significantly associated with postoperative HRQOL (Test 2). **(B)** In Test 2, pain during exertion was significantly associated with HRQOL. HRQOL, health-related quality of life.

Of the above seven items that were significantly associated with HRQOL in Test 2, four items that were considered to have a large clinical impact on HRQOL were set as independent variables (state anxiety in Test 1, HRQOL in Test 1, pain at rest in Test 2, and pain during exertion in Test 2). Multiple regression analysis revealed that state anxiety in Test 1 (*B* = −0.361, *p* = 0.004, Figure 3A) and pain on exertion in Test 2 (*B* = −0.353, *p* = 0.049) were significant predictors of HRQOL in Test 2. The analysis also revealed that pain during exertion in Test 2 was significant predictor of HRQOL in Test 2 (Figure 3B). In addition, we tested the associations between preoperative anxiety with HRQOL at 1 month and 3 months after surgery. Multiple regression analysis showed that state (*B* = 0.039, *p* = 0.836) and trait anxiety (*B* = 0.048, *p* = 0.797) in Test 1 were no association for HRQOL in Test 3. Similarly, state (*B* = −0.077, *p* = 0.645) and trait anxiety (*B* = 0.282, *p* = 0.099) in Test 1 were no association for HRQOL in Test 4.

To test the effects on types of surgery, two-way ANOVA was conducted with the surgical approach (VATS or Open chest) and lung resection mode (Wedge or other) as factors for postoperative HRQOL. The analysis showed that there were no significance on main effects of surgical approach (*F*_1,47_ = 1.051, *p* = 0.310, *η*^2^ = 0.022), lung resection mode (*F*_1,47_ = 3.469, *p* = 0.069, *η*^2^ = 0.069), and the interaction (*F*_1,47_ = 0.563, *p* = 0.457, *η*^2^ = 0.012) in the HRQOL at Test 2. Similarly, in the HRQOL at Test 3, there were no significance on main effects of surgical approach (*F*_1,40_ = 0.945, *p* = 0.337, *η*^2^ = 0.158), lung resection mode (*F*_1,40_ = 0.073, *p* = 0.788, *η*^2^ = 0.058), and the interaction (*F*_1,40_ = 0.009, *p* = 0.925, *η*^2^ = 0.001). In the HRQOL at Test 4, there were no significance on main effects of surgical approach (*F*_1,36_ = 0.299, *p* = 0.588, *η*^2^ = 0.008), lung resection mode (*F*_1,36_ = 0.046, *p* = 0.832, *η*^2^ = 0.001), and the interaction (*F*_1,36_ = 0.046, *p* = 0.832, *η*^2^ = 0.001).

## Discussion

The trend that HRQOL is lowest at the time of discharge and recovers to some extent after several months has been reported in previous studies (Stigt et al., 2013; Ichimura et al., 2021a,b), and the same trend was observed in the present study. Related to the trend of HRQOL, pain decreased after 3 months. Furthermore, the study showed quantitatively that preoperative “state anxiety” was a predictor of HRQOL at discharge, using a clinical assessment of anxiety.

In the single regression analysis, several background factors were associated with postoperative HRQOL (test 2). Comorbid stroke has been reported to increase the pain response threshold (Vestergaard et al., 1995), which may have had some influence on this study. This suggests that preoperative state anxiety had the greatest effect on postoperative HRQOL. In the postoperative period (test 2), pain during exertion was directly related to postoperative HRQOL. This suggests that pain assessment is a major part of HRQOL.

Why did preoperative state anxiety affect postoperative HRQOL? There are at least physiological and cognitive functional reasons for this. High levels of anxiety can lead to negative physiological symptoms such as increased blood cortisol levels, increased blood pressure and heart rate, delayed wound healing, decreased immune response, and increased risk of infection (Scott, 2004). High anxiety can also adversely affect the induction of anesthesia and impair postoperative recovery (Kiecolt-Glaser et al., 1998; Kain et al., 2000). These physiological changes during surgery as a result of high anxiety may reduce postoperative HRQOL. On the other hand, emotions associated with anxiety are processed in the amygdala and affect several brain regions (Ghasemi et al., 2022; Kami et al., 2022). In particular, the hippocampus is more likely to retain emotion-related memories (Mathis and Lecourtier, 2017). Anxiety is also known to cause cognitive distortions and non-adaptive behavior (Alladin and Amundson, 2016). When anxiety is high, it may evoke stronger negative emotions than necessary in response to the negative event of surgery, resulting in it being retained as an intense negative memory. It is possible that cognitive function was also associated with lower postoperative HRQOL.

The current study has a number of limitations. First, this study focused on postoperative HRQOL in lung cancer surgery, but we did not investigate it in surgery other than lung cancer. In the future, it is necessary to investigate in other than lung cancer and verify whether the results of the present study can be generalized to all surgical procedures. Second, because the STAI and HRQOL assessments are self-report measures, various awareness biases may be associated with the results. This method is subject to patient subjectivity and may reflect social desirability and other factors in the responses. It is important to measure the patient’s brain using MRI before and after surgery. This should provide a neuroscientific basis for predicting postoperative HRQOL and an indicator that is as free from subjective bias as possible. From this validation, it may be possible to rigorously establish a method to reduce preoperative anxiety and improve postoperative HRQOL. Finally, the study did not involve a psychiatric clinical interview and may have included patients who originally had an anxiety disorder. Future research should also look more closely at the original mental characteristics of the patients.

This study offers the possibility that preoperative state anxiety may be a predictor of postoperative HRQOL immediately after surgery. Improving state anxiety through psychotherapy, such as mindfulness and music therapy, before surgery may improve postoperative HRQOL (Khoury et al., 2013; Bradt et al., 2016; Hofmann et al., 2017; Carpenter et al., 2018; Felsch and Kuypers, 2022). Empirical studies that use psychotherapy to manipulate preoperative anxiety are important.

## Data availability statement

The original contributions presented in the study are included in the article/Supplementary material, further inquiries can be directed to the corresponding author.

## Ethics statement

The studies involving human participants were reviewed and approved by the Ethics Committee of Showa University School of Medicine. The patients/participants provided their written informed consent to participate in this study.

## Author contributions

MH and YM designed the study. ST, MO, SO, YT, KS, SU, and AK recruited the patients and conducted the experiment. ST, MH, YM, and MI analyzed data. MH was major contributor to writing the manuscript. All authors contributed to the article and approved the submitted version.

## Funding

ST was supported by JSPS KAKENHI (Grant Number: 20K17758).

## Conflict of interest

The authors declare that the research was conducted in the absence of any commercial or financial relationships that could be construed as a potential conflict of interest.

## Publisher’s note

All claims expressed in this article are solely those of the authors and do not necessarily represent those of their affiliated organizations, or those of the publisher, the editors and the reviewers. Any product that may be evaluated in this article, or claim that may be made by its manufacturer, is not guaranteed or endorsed by the publisher.
