# Supplementary material for: Preoperative state anxiety predicts postoperative health-related quality of life: A prospective observational study on patients undergoing lung cancer surgery
Source: Front Psychol. 2023 Apr 11;14:1161333. doi: 10.3389/fpsyg.2023.1161333 (PMC10126259; doi:10.3389/fpsyg.2023.1161333)
Supplement: Supplementary file 1 [file Data_Sheet_1.pdf]

Supplementary Information

Preoperative state anxiety predicts postoperative quality of life: A prospective observational study on patients undergoing lung cancer surgery

Shinnosuke Takamiya, Motoyasu Honma, Yuri Masaoka, Momoka Okada, Shinichi Ohashi, Yoko Tanaka, Kosuke Suzuki, Shugo Uematsu, Akihiko Kitami, Masahiko Izumizaki

Supplementary Tables 1-2

S1 Table. Anxiety, HRQOL, and pain.

	Test	Cases (N)	Min	Max	Mean	S.D.
State anxiety						
	1	51	27	60	40.14	6.85
	2	48	29	53	39.81	6.02
	3	44	28	50	38.80	5.81
	4	41	27	51	41.95	5.44
Trait anxiety						
	1	51	34	60	46.43	5.59
	3	48	29	55	44.56	5.25
	3	44	32	62	43.50	6.41
	4	41	34	56	44.76	5.74
HRQOL						
	1	51	0.552	1	0.896	0.109
	2	51	0.408	1	0.764	0.133
	3	44	0.389	1	0.808	0.137
	4	40	0.704	1	0.900	0.095
Pain						
At rest	2	51	0	8	1.88	2.30
At move	2	51	1	9	4.20	2.41
At rest	3	44	0	10	1.77	2.34
At move	3	44	0	10	3.18	2.47
At rest	4	40	0	10	0.62	1.63
At move	4	40	0	9	0.95	1.72

S.D.: Standard deviation, HRQOL: Health-related quality of life.

S2 Table. Association of perioperative HRQOL with clinical factors.

	B	S.E.	P value
Sex (female ratio)	0.025	0.039	0.525
Age	0	0.002	0.838
Stature (cm)	-0.004	0.002	0.053
Body weight (kg)	-0.001	0.002	0.458
Tumor diameter (mm)	-0.003	0.002	0.112
CCI (0)	-0.016	0.038	0.672
Comorbidities (-)			
BA	0.068	0.079	0.394
Af	0.09	0.096	0.352
Hypertension	0.045	0.039	0.249
Interstitial pneumonia	-0.068	0.135	0.619
Ischemic heart disease	0.104	0.079	0.190
Stroke	0.311	0.128	0.019
Diabetes mellitus	-0.048	0.051	0.357
COPD	-0.045	0.058	0.443
Smoking index	< 0.0001	< 0.0001	0.246
Clinical stage category (<= I)	0.065	0.051	0.210
Surgical approach (VATS)	-0.014	0.04	0.732
Lung resection mode (<= Wedge)	0.071	0.042	0.097
Postoperative adverse event, present (<= Grade 1)	0.006	0.055	0.919
Operating Time (min)	0	0	0.095
Volume of blood loss (ml)	< 0.0001	0	0.480
Wound length (cm)	-0.005	0.005	0.286
Postoperative hospital stay (days)	0.004	0.007	0.569
Postoperative treatment	0.089	0.041	0.037
State anxiety			

Test 1	-0.009	0.002	0.001
Test 2	-0.003	0.003	0.371
Test 3	-0.001	0.003	0.790
Test 4	-0.005	0.004	0.155
Trait anxiety			
Test 1	-0.002	0.003	0.618
Test 2	-0.002	0.004	0.564
Test 3	-0.004	0.003	0.203
Test 4	0.004	0.004	0.253
HRQOL (EQ-5D-5L)			
Test 1	0.352	0.168	0.041
Test 2			
Test 3	0.420	0.132	0.003
Test 4	0.323	0.217	0.144
Pain			
Test 2 (at rest)	-0.020	0.008	0.014
Test 2 (at move)	-0.025	0.007	< 0.0001
Test 3 (at rest)	-0.005	0.009	0.524
Test 3 (at move)	-0.011	0.008	0.192
Test 4 at rest)	-0.011	0.013	0.377
Test 4 (at move)	-0.020	0.012	0.098

S.E.: Standard error, HRQOL: Health-related quality of life, CCI: Charlson comorbidity index, BA: bronchial asthma, Af: atrial fibrillation, COPD: Chronic Obstructive Pulmonary Disease.
